# Effect of Dialysis Modality on Mortality and Complications in Cardiovascular Surgery: Insights from a National Retrospective Cohort Study

**DOI:** 10.34067/KID.0000000701

**Published:** 2025-01-16

**Authors:** Ankur Shah, Osama El Shamy, Christina A. Raker, Jeffrey Perl, Susie Hu

**Affiliations:** 1 Warren Alpert Medical School of Brown University, Providence, Rhode Island; 2 Division of Kidney Disease and Hypertension, Department of Medicine, Rhode Island Hospital, Providence, Rhode Island; 3 Division of Renal Diseases and Hypertension, George Washington University, Washington, DC; 4 Biostatistics, Epidemiology, Research Design, and Informatics Core, Rhode Island Hospital, Providence, Rhode Island; 5 Division of Nephrology, St. Michael's Hospital, Unity Health, University of Toronto, Toronto, Ontario, Canada

**Keywords:** dialysis, peritoneal dialysis

## Abstract

**Key Points:**

- Maintaining peritoneal dialysis during the perioperative period may confer benefits over hemodialysis, including lower hospital charges and improved patient outcomes.
- Peritoneal dialysis is associated with lower in-hospital mortality compared with hemodialysis in patients undergoing cardiac surgery.

**Background:**

Patients receiving maintenance dialysis face high mortality and complication rates after cardiovascular (CV) surgery. With the growing utilization of peritoneal dialysis (PD), it is important to understand the effect of modality (PD versus center-based hemodialysis) on outcomes after CV surgery.

**Methods:**

This retrospective cohort study used data from the National Inpatient Sample (2016–2020) to compare outcomes of PD and hemodialysis patients undergoing coronary artery bypass grafting and surgical valve procedures. Multivariable logistic regression, negative binomial regression, and linear regression models were used, adjusting for demographic factors, comorbidities, and hospital characteristics. The primary outcome was in-hospital mortality, and secondary outcomes included prolonged ventilation, length of stay, and hospital charges.

**Results:**

A total of 30,155 patients were included in the study, with 28,015 (92.9%) receiving hemodialysis and 2140 (7.1%) receiving PD. Compared with hemodialysis, PD was associated with lower in-hospital mortality (odds ratio, 0.61; 95% confidence interval [CI], 0.38 to 0.97), reduced prolonged ventilation (odds ratio, 0.51; 95% CI, 0.32 to 0.81), shorter length of stay (incidence rate ratio, 0.85; 95% CI, 0.80 to 0.91), and decreased hospital charges (mean difference, −$87,172 USD; 95% CI, −$113,523 to −$60,820) compared with hemodialysis. Among PD patients, postoperative transition to hemodialysis was associated with worse outcomes.

**Conclusions:**

Maintaining PD during the perioperative period may confer benefits over hemodialysis, including lower hospital charges and improved patient outcomes. Careful consideration of dialysis modality in the management of CV surgery patients is needed to optimize clinical outcomes and reduce health care costs.

## Visual Abstract

**Figure:**
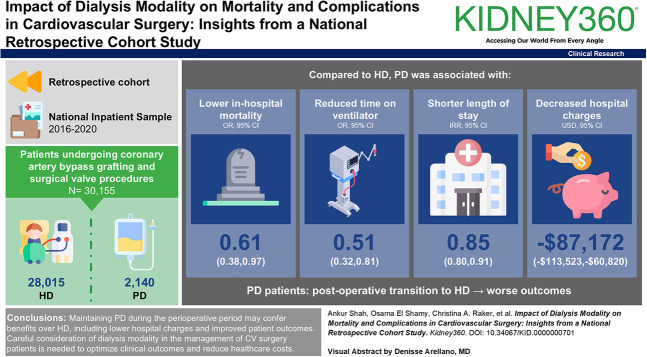

## Introduction

Patients receiving maintenance dialysis have a twenty-fold higher risk of cardiovascular (CV) mortality compared with the general population, and many will require CV surgery during their lifetime.^1^ In addition, compared with nondialysis patients, dialysis patients undergoing coronary artery bypass grafting (CABG) or surgical valve replacement have up to a four-fold higher risk of in-hospital mortality, increased complication rates, and prolonged lengths of stay.^2–8^ With recent policy changes, including the Advancing American Kidney Health initiative and the ESKD Treatment Choices model, which uses a payment adjustment system that incentivizes home dialysis utilization and transplant rates while imposing penalties for underperformance in the United States, aiming to increase the utilization of home dialysis modalities,^9,10^ a growing number of patients with ESKD needing cardiac surgery are being treated with peritoneal dialysis (PD).^11,12^

Historically, concerns have been raised that PD patients may be at higher risk for complications postcardiac surgery compared with those receiving center-based hemodialysis, including inadequate ultrafiltration and volume control, increased bleeding risks, higher rates of pericardial effusions, and higher rates of mediastinitis and sternal wound infections.^13^ This has led to PD patients frequently being electively converted to hemodialysis in the perioperative period.^11,13–15^ However, the current evidence comparing outcomes between PD and hemodialysis patients after cardiac surgery is limited and conflicting, with some single-center studies showing no difference in mortality^8,16–18^ and a lower composite outcome of in-hospital events (death, cardiac arrest, pericardial effusion, and sternal wound infections) in PD patients,^13^ while others found higher operative mortality in older PD patients.^8,15^ Variations in prescription patterns and treatment implementation postoperatively can have significant effects on these studies' outcomes. Furthermore, the reasons for and outcomes associated with postoperative conversion from PD to hemodialysis remain poorly characterized.^11^

The limited and conflicting evidence, coupled with the growing prevalence of PD, highlights the need for further investigation into the impact of dialysis modality on cardiac surgery outcomes. Evidence to support PD continuation versus conversion to hemodialysis is needed to best inform the clinical team when determining postoperative dialysis modality utilization for the individual patient.

This study aims to compare the outcomes of PD and hemodialysis patients undergoing CABG and/or surgical valve replacement. Using data from the National Inpatient Sample (NIS) from 2016 to 2020, we set out to compare postoperative morbidity and mortality in patients with ESKD stratified by dialysis modality. We also examine differences in hospitalization duration, hospital charges, and the effect of modality transitions from PD to hemodialysis.

## Methods

### Study Design

This was a retrospective cohort study using the NIS from 2016 to 2020. The NIS is sponsored by the Agency for Healthcare Research and Quality as part of the Healthcare Cost and Utilization Project and is the largest publicly available all-payer inpatient database in the United States. The NIS samples over 7 million community hospital stays each year using a complex sampling design to enable national estimates. Rehabilitation and long-term acute care facilities are excluded from sampling. The Lifespan Health System Institutional Review Board (IRB) acknowledged our study submission, but noted that IRB oversight was not required given that the NIS data met the criteria for research not involving human participants.

### Population/Exposure

The exposure of interest was dialysis status stratified by modality (hemodialysis versus PD). Dialysis patients were identified by the presence of International Classification of Diseases, 10th revision (ICD-10) diagnosis codes for ESKD (N18.6), as well as the absence of ICD-10-clinical modification codes for AKI (N17.x) and the presence of ICD-10 procedure coding system for hemodialysis (5A1D00Z, 5A1D60Z, 5A1D70Z, 5A1D80Z), continuous KRT (5A1D90Z), or PD (3E1M39Z).

The study population comprised adult patients aged 18 years or older undergoing cardiac surgery identified by ICD-10 procedure coding system (Supplemental Table 1). Patients with missing data on age, sex, race, or outcomes were excluded. As a secondary aim, we restricted the population to those receiving PD only and defined a secondary exposure of hemodialysis after PD. This was defined using time to procedure data with hemodialysis after PD being defined as sole receipt of PD preoperatively with procedure code for receipt of hemodialysis on or after the day of surgery.

### Covariates

We collected information on demographic, clinical, and hospital-level covariates. Demographic factors included age, sex, race/ethnicity, and primary method of payment. Clinical factors included burden of chronic coexisting illnesses measured by the Charlson comorbidity index, along with pre-existing diabetes, high BP, congestive heart failure (CHF), cerebrovascular disease, and cancer, identified using ICD-10-clinical modification codes (see Supplemental Table 2). Hospital-level factors included location (rural versus urban), geographic region, teaching status, and number of beds.

### Outcomes

The primary outcome was in-hospital mortality, defined as death occurring during the index hospitalization. Secondary outcomes included prolonged ventilation, defined as need for mechanical ventilation >96 hours, total hospital charges, and total hospital length of stay.

### Statistical Methods

Demographic and clinical characteristics were summarized by dialysis modality. The primary analysis used multivariable logistic regression to evaluate the association between dialysis modality and in-hospital mortality, adjusting for demographics, comorbid conditions, and hospital factors as potential confounders. For secondary outcomes, we used multivariable logistic regression for prolonged ventilation, negative binomial regression for length of stay, and linear regression for hospital charges. Similar covariate adjustments were used. Survey-specific analytic procedures incorporated discharge weights, sampling units, and sampling strata to generate nationally representative estimates for all analyses. This approach accounts for clustering within hospitals. Results were reported as adjusted odds ratios (ORs), incidence rate ratios (IRRs), and mean differences (MDs) with 95% confidence intervals (CIs). We additionally evaluated the risk factors for and effect of modality transition using simple logistic, negative binomial, and linear regression as appropriate. Multivariable adjustment was not performed because of the low number of events. Prespecified subgroup and exploratory analyses were planned by categories of cardiac procedures, single versus multiple valve replacements, surgery on hospital day 0 or 1 compared with later in hospitalization, as well as receipt cardiopulmonary bypass. All *P* values were two-sided, with *P* < 0.05 considered statistically significant. Analyses were conducted using Stata 17 (College Station, TX).

### Ethics Approval

The protocol for this research project was approved by the Lifespan IRB and conforms to the provisions of the Declaration of Helsinki.

## Results

**Figure 1:**
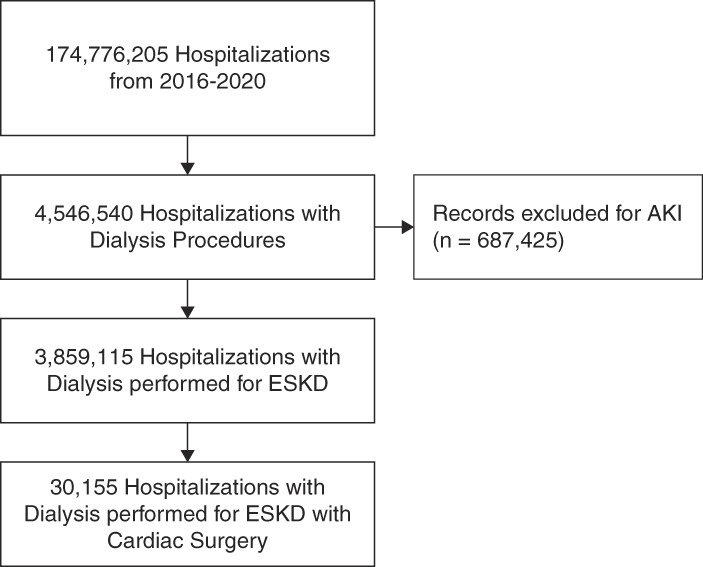
Study population.

A total of 30,155 patients were included in the study, with 28,015 (92.9%) receiving hemodialysis and 2140 (7.1%) receiving PD (Figure 1). Most of the patients in both groups were aged 55 years or older (hemodialysis: 75.9%, PD: 74.7%). The proportion of female patients was slightly lower in the PD group (28.7%) compared with the hemodialysis group (31.8%). A higher proportion of patients in the PD group (57.7%) were White compared with the hemodialysis group (44.0%). Conversely, the hemodialysis group had a higher proportion of Black (24.2% versus 14.2%) and Hispanic (17.2% versus 14.2%) patients. The prevalence of CHF was higher in the hemodialysis group (67.0%) compared with the PD group (56.8%). Diabetes was more prevalent in the PD group (72.4%) than in the hemodialysis group (68.5%). The prevalence of other comorbidities, including cerebrovascular disease, hypertension, and cancer, was similar between the two groups. The remainder of baseline characteristics are found in Table 1.

**Table 1:** Baseline characteristics by dialysis modality

Baseline Characteristics			*P* Value
Characteristic	Hemodialysis	PD	
Total	28,015 (92.9)	2140 (7.1)	
**Age, yr**			0.114
Mean (SD)	61.3 (11.4)	61.7 (10.4)	
18–44	2215 (7.9)	100 (4.7)	
45–54	4865 (17.4)	415 (19.4)	
55–64	9090 (32.4)	700 (32.7)	
65–74	8555 (30.5)	715 (33.4)	
≥75	3275 (11.7)	210 (9.8)	
Female sex	8910 (31.8)	6.15 (28.7)	0.191
**Race**			<0.001
Asian	1885 (6.7)	135 (6.3)	
Black	6795 (24.2)	305 (14.2)	
Hispanic	4830 (17.2)	305 (14.2)	
Other	1320 (4.7)	90 (4.2)	
White	12,335 (44.0)	1235 (57.7)	
**CCMI**			0.424
2	960 (3.4)	105 (4.9)	
3	2695 (9.6)	210 (9.8)	
4	5325 (19.0)	415 (19.4)	
5	19,035 (68.0)	1410 (65.9)	
**Comorbidities**			
CHF	18,785 (67.0)	1215 (56.8)	<0.001
CEVD	3620 (12.9)	270 (12.6)	0.858
Hypertension	27,190 (97.0)	2075 (96.9)	0.912
Diabetes	19,180 (68.5)	1550 (72.4)	0.100
Cancer	505 (1.8)	60 (2.8)	0.167
**Hospital bed size**			0.050
Small	2785 (10.0)	145 (6.8)	
Medium	6395 (22.8)	440 (20.6)	
Large	18,835 (67.2)	1555 (72.7)	
**Hospital location**			0.610
Rural	410 (1.5)	25 (1.2)	
Urban	27,605 (98.5)	2115 (98.8)	
**Teaching status**			0.673
Nonteaching	4080 (14.6)	295 (13.8)	
Teaching	23,935 (85.4)	1845 (86.2)	
**Hospital region**			0.183
Northeast	4395 (15.7)	265 (12.4)	
Midwest	6115 (21.8)	465 (21.7)	
South	11,165 (39.9)	845 (39.5)	
West	6340 (22.6)	565 (26.4)	
**Primary payer**			<0.001
Medicare	20,235 (72.3)	1495 (70.2)	
Medicaid	2585 (9.2)	105 (4.9)	
Private	4345 (15.5)	485 (22.8)	
Self-pay or no charge	295 (1.1)	15 (0.7)	
Other	540 (1.9)	30 (1.4)	
CV surgeries	28,015	2140	
Any CABG	21,795 (77.8)	1865 (87.1)	<0.001
Any valve surgeries	9790 (34.9)	550 (25.7)	<0.001
Off pump	5160 (23.6)	310 (14.5)	0.063
Combined CABG+valve	3570 (12.7)	275 (12.9)	<0.001

Data presented as weighted mean (SD) for continuous variables and *n* (%) for categorical variables. Percentages may not sum to 100% because of rounding. Some patients may have undergone both CABG and valve surgeries, so the total number of CABG and valve surgeries may exceed the total number of CV surgeries. CABG, coronary artery bypass grafting; CCMI, Charlson comorbidity index; CEVD, cerebrovascular disease; CHF, congestive heart failure; CV, cardiovascular; PD, peritoneal dialysis.

CABG was the most common procedure performed, accounting for 23,660 (78.5%) of all surgeries, with 21,795 in the hemodialysis group and 1865 in the PD group. Valve surgeries were performed in 10,340 (34.3%) patients, with 9790 in the hemodialysis group and 550 in the PD group. Combined CABG and valve surgery was performed in 3845 (12.8%) patients, with 3570 in the hemodialysis group and 275 in the PD group (Table 1). Of the patients who received hemodialysis before surgery, 99.3% (*n*=28,015) remained on hemodialysis postoperatively. Among patients who were on PD before surgery (*n*=2140), 1615 (75.5%) remained on PD postoperatively, while 525 (24.5%) switched to hemodialysis. Continuous KRT was used in 2305 (7.6%) patients postoperatively, with 2150 (7.7%) in the hemodialysis group and 155 (7.2%) in the PD group (Table 2).

**Table 2:** Dialysis modalities received by initial modality

Modality	Preoperative Hemodialysis, *n* (%)	Preoperative PD, *n* (%)
Postoperative hemodialysis	27,830 (99.3)	525 (24.5)
Postoperative PD	185 (0.7)	1615 (75.5)
Postoperative CKRT	2150 (7.7)	155 (7.2)

CKRT, continuous KRT; PD, peritoneal dialysis.

Patients in the PD group experienced better outcomes compared with those in the hemodialysis group across all measured variables (Table 3). The unadjusted in-hospital mortality was lower in the PD group (4.4% versus 7.8%; OR, 0.55; 95% CI, 0.35 to 0.88), and this difference remained significant after adjustment (OR, 0.61; 95% CI, 0.38 to 0.97). Similarly, the incidence of prolonged ventilation was lower in the PD group (4.7% versus 9.7%; unadjusted OR, 0.45; 95% CI, 0.29 to 0.71; adjusted OR, 0.51; 95% CI, 0.32 to 0.81). The average length of stay was shorter in the PD group (13.6 versus 17.1 days; unadjusted IRR, 0.79; 95% CI, 0.74 to 0.85; adjusted IRR, 0.85; 95% CI, 0.80 to 0.91). Finally, total charges were significantly lower in the PD group (mean $307,072 versus $407,556; unadjusted MD, −$100,484; 95% CI, −$124,773 to −$76,195; adjusted MD, −$87,172; 95% CI, −$113,523 to −$60,820). These findings were consistent among procedure types (Supplemental Table 3).

**Table 3:** Outcomes^e^ and models by modality

Modality	Mortality^a^			Prolonged Ventilation^a^				Length of Stay (d)^b^				Total Charges ($)^c^			
	*n* (%)	Unadjusted OR (95% CI)	Adjusted^d^ OR (95% CI)	*n* (%)	Unadjusted OR (95% CI)	Adjusted^d^ OR (95% CI)	Mean (SD)		Unadjusted IRR (95% CI)	Adjusted^d^ IRR (95% CI)	Mean (SD)		Unadjusted MD (95% CI)	Adjusted^d^ MD (95% CI)	
Hemodialysis	2170 (7.8)	1.00 (Ref)	1.00 (Ref)	2730 (9.7)	1.00 (Ref)	1.00 (Ref)	17.1 (17.2)		1.00 (Ref)	1.00 (Ref)	407,556 (434,930)		0 (Ref)	0 (Ref)	
PD	95 (4.4)	0.55 (0.35 to 0.88)	0.61 (0.38 to 0.97)	100 (4.7)	0.45 (0.29 to 0.71)	0.51 (0.32 to 0.81)	13.6 (9.8)		0.79 (0.74 to 0.85)	0.85 (0.80 to 0.91)	307,072 (221,832)		−100,484 (−124,773 to 76,195	−87,172 (−113,523 to 60,820)	

CI, confidence interval; IRR, incidence rate ratio; MD, mean difference; OR, odds ratio; PD, peritoneal dialysis; Ref, reference group.

a OR with 95% CI from logistic regression.

b IRR with 95% CI from negative binomial regression.

c Mean difference with 95% CI from linear regression.

d Adjusted for age, sex, race, Charlson comorbidity index, congestive heart failure, cerebrovascular disease, hypertension, diabetes, cancer, hospital bed size, hospital location, urban/rural, teaching versus nonteaching, payer, coronary artery bypass grafting, and/or valve surgery.

e Prolonged ventilation was defined as mechanical ventilation >96 hours. Length of stay is reported in days. Total charges are reported in US dollars.

**Figure 2:**
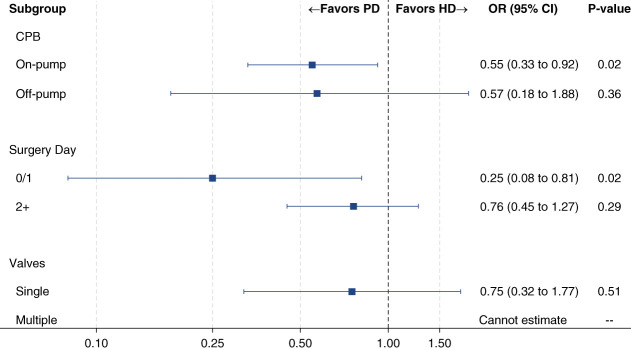
**Mortality subgroups.** CI, confidence interval; CPB, cardiopulmonary bypass; HD, hemodialysis; OR, odds ratio; PD, peritoneal dialysis.

A number of subgroups and exploratory analyses were evaluated in this study (Figure 2). Findings were largely consistent with overlap of CIs for mortality, length of stay, prolonged ventilation, and total charges in subgroups of on-pump and off-pump surgery, hospital day 0 or 1 versus 2+ surgery, and single and multiple valve replacements (Supplemental Figures 1–3).

In a subgroup analysis of patients who either underwent PD only or transitioned from PD to hemodialysis, those who transitioned had significantly worse outcomes (Table 4). Patients who transitioned from PD to hemodialysis had 2.66 times higher odds of prolonged ventilation (OR, 2.66; 95% CI, 1.06 to 6.65), $122,166 higher total charges on average (95% CI, 62,193 to 182,139), and a 57% longer length of stay (IRR, 1.57; 95% CI, 1.33 to 1.80) compared with those who underwent PD only. Although the odds of in-hospital mortality were 2.34 times higher in the transition group, this difference did not reach statistical significance (OR, 2.34; 95% CI, 0.90 to 6.01). Risk factors for conversion to hemodialysis after surgery in PD patients included CHF, peripheral vascular disease, midwestern residence, combined CABG/valve surgery, and cardiopulmonary bypass (Table 5).

**Table 4:** Outcomes and models by modality transition

Outcome	PD Only	Hemodialysis after PD	Unadjusted Effect Size^a^
Mortality, *n* (%)	55 (3.4)	40 (7.6)	2.34 (0.90 to 6.01)^b^
Prolonged ventilation, *n* (%)	55 (3.4)	45 (8.6)	2.66 (1.06 to 6.65)^b^
Length of stay, mean (SD)	11.98 (8.50) d	18.51 (11.70) d	1.57 (1.33 to 1.80)^c^
Total charges, mean (SD)	$276,893 ($188,167)	$399,060 ($283,378)	$122,166 ($62,193 to $182,139)^d^

PD, peritoneal dialysis.

a Reference group: PD only.

b Odds ratio with 95% confidence interval from logistic regression.

c Incidence rate ratio with 95% confidence interval from negative binomial regression.

d Adjusted mean difference with 95% confidence interval from linear regression.

**Table 5:** Risk factors for hemodialysis transfer in peritoneal dialysis

Risk Factor	OR (95% CI)
**Age**	1.00 (Ref)
18–44	0.67 (0.24 to 1.89)
45–54	0.55 (0.20 to 1.50)
55–64	0.56 (0.21 to 1.51)
65–74	0.66 (0.21 to 2.08)
Older than 75	
Female	1.12 (0.70 to 1.79)
**Race**	
Asian/Pacific Islander	1.09 (0.46 to 2.57)
Black	1.02 (0.52 to 1.99)
Hispanic	1.11 (0.58 to 2.12)
Other	1.04 (0.32 to 3.34)
White	1.00 (Ref)
CCMI (per point)	1.19 (0.88 to 1.59)
CHF	1.83 (1.18 to 2.86)
Peripheral vascular disease	2.00 (1.08 to 3.71)
Hypertension	1.09 (0.29 to 4.03)
Diabetes	1.06 (0.65 to 1.73)
Need for pressors	1.56 (0.52 to 4.70)
Teaching hospital	1.05 (0.56 to 1.98)
**Hospital region**	
Northeast	1.00 (Ref)
Midwest	2.26 (1.01 to 5.06)
South	1.25 (0.58 to 2.69)
West	1.22 (0.55 to 2.72)
**Hospital bed size**	
Small	1.00 (Ref)
Medium	0.81 (0.31 to 2.10)
Large	1.09 (0.47 to 2.54)
**Hospital location**	
Rural	1.00 (Ref)
Urban	1.30 (0.14 to 11.87)
**Payer**	
Medicare	1.00 (Ref)
Medicaid	0.66 (0.21 to 2.01)
Private Insurance	0.72 (0.41 to 1.27)
Other	0.56 (0.06 to 4.84)
Combined CABG/valve	1.94 (1.06 to 3.55)
On-pump	2.43 (1.13 to 5.24)

CABG, coronary artery bypass grafting; CCMI, Charlson comorbidity index; CHF, congestive heart failure; CI, confidence interval; OR, odds ratio; Ref, reference group.

## Discussion

In this study, we found that PD was associated with lower in-hospital mortality, reduced duration of mechanical ventilation, shorter length of stay, and decreased hospital charges compared with hemodialysis in patients undergoing cardiac surgery. We also found that among PD patients, postoperative transition to hemodialysis was associated with prolonged ventilation, increased length of stay, and total hospital charges. Our findings are important because they suggest that PD may be a safe and potentially preferable dialysis modality for patients requiring cardiac surgery, and that efforts should be made to maintain PD in the perioperative period.

The prevalence of coronary heart disease is approximately 40% in patients on dialysis.^19^ Moreover, rates of CV calcification range from as low as 23% to as high as 57.5%^20^ It is therefore imperative that a significant percentage of dialysis patients will require some form of cardiac surgery. Given the growing incidence and prevalence of PD utilization,^12^ as well as the emphasis on honoring patient modality selection, our study provides further information to help guide the clinical decision-making process.

The existing literature comparing PD and hemodialysis outcomes after cardiac surgery is limited and conflicting. While some single-center studies have shown no difference in mortality, others have found higher death rates in older PD patients, and reduced composite in-hospital events in PD patients. The unadjusted mortality of 4.4% for the PD patients in our cohort was in the low end of the widely ranging values (2%–58%) observed in other studies and to a lesser degree for the hemodialysis group (7.8% versus 1.6%–15%). Li *et al.* and Zong *et al.* found their excessively high PD mortality attributed primarily to sepsis.^8,15^ We found a lower PD length of stay of 14 days in comparison with 17 days in the hemodialysis group, although other studies reported highly variable findings—hospital stay ranging 1–36 days for PD versus 8–40 days for hemodialysis. This was also true for intensive care unit stay (1–14 days for PD versus 1–9 days for hemodialysis).^8,13,15,17^ These discrepant findings may be due to small sample sizes, differences in patient selection, PD prescription patterns, residual kidney function, perioperative management, as well as residual confounding. Our study addresses many of these limitations by using a large, nationally representative sample and adjusting for a wide range of potential confounders. The lower morbidity and mortality observed with PD in our study is biologically plausible and may be related to several factors. First, PD is a continuous therapy that provides more steady fluid and solute removal, reducing the risk of rapid changes in volume status and electrolyte disturbances that can be seen with hemodialysis. Second, PD is performed without the need for anticoagulation, which may decrease bleeding complications. Third, PD avoids the need for central venous access and potential infectious complications.

Approximately 25% of PD patients in our cohort were converted to hemodialysis postoperatively, and this transition was associated with worse outcomes. Prior studies reported PD to hemodialysis conversion rates ranging from 5.5% to 26% postcardiac surgery.^13,16^ Reasons for conversion included catheter malfunction, pericardio-peritoneal shunting, clinician choice, and hemodynamic instability.^13^ While the reasons for conversion in our cohort is unknown, we suspect that reasons for conversion are similar. Nevertheless, our findings suggest that every effort should be made to maintain prevalent PD patients on PD in the postoperative period. Maintenance of PD allow for avoidance of hemodialysis catheters and their associated complications. This requires inpatient protocols, availability of PD cyclers and supplies, and the presence of PD-trained clinical staff to optimize the management of these patients.

There are clinical situations where the transition from PD to hemodialysis is medically necessarily, and it is important that our findings not be misconstrued as advocating for PD continuation regardless of clinical scenario. Insufficient ultrafiltration, catheter mechanical problems, insufficient clearance of uremic toxins, and peritonitis are among potential causes for a transient transition to hemodialysis in the postoperative period. Patients transitioned from PD to hemodialysis postsurgery may represent a clinically unstable population, which could confound the observed associations. This is acknowledged as a limitation. The unavailability of the clinical staff and support needed to provide inpatient PD, as well as lack of acute rehabilitation or nursing home facilities providing PD care, are also obstacles that can result in the transition to hemodialysis postoperatively in some medical institutions. Arranging for a PD patient to have their cardiac surgery performed in a hospital that does provide inpatient PD services is a solution to this issue, provided it is within a reasonable distance from the patient.

In 2021, the total Medicare fee-for-service expenditure for inpatient patients with ESKD was $11 billion, $2.6 billion of which were for CV admissions.^12^ We found that the continuation of PD in prevalent PD patients postcardiac surgery resulted in a mean reduction in hospital charges of $122,166. This figure does not include the cost savings to the health care system and payors if these patients who were transitioned to hemodialysis or discharged on hemodialysis then require retraining and reinitiation of PD. The continuation of PD inpatient is also more cost-effective because of the reduced facility overhead expenses, staffing requirements and labor costs. Given that ESKD spending on Medicare fee-for-service comprised 6.8% of total Medicare spending in 2021, a clinical decision that would allow for the honoring of patient modality selection, cost-savings for the health care system, all while not compromising patients' well-being, is a logical one.

Our study has many strengths. This is a large, nationally representative dataset with detailed patient and hospital-level data and included a multipayer cohort. Adjustments were made for multiple potential confounders and included the assessment of several clinically relevant outcomes. Our study also has several limitations. First, as with all observational studies, residual confounding is possible despite adjustment for multiple variables. We lacked data on patients' dialysis vintage, details regarding the dialysis prescription, urea kinetics, and residual kidney function, which may affect outcomes. Second, we could not determine the reasons for postoperative conversion from PD to hemodialysis, which may have provided insights into how to avoid this complication. In addition, the study period included the coronavirus disease 2019 pandemic (2019–2020), which may have influenced outcomes due to shifts in resource allocation, including changes in surgical practices and shortages of hemodialysis machines. Finally, we only assessed in-hospital outcomes, and longer-term follow-up is needed to evaluate the effect of dialysis modality on postdischarge outcomes.

Future studies should evaluate the long-term outcomes of PD and hemodialysis patients after cardiac surgery and investigate strategies to reduce the risk of postoperative complications in this high-risk population. Studies that can capture the reasons for postoperative conversion from PD to hemodialysis and assess the effect of PD prescription and urea kinetics on outcomes would be particularly informative. Incorporation of more granular clinical data, including intraoperative parameters and postoperative complications, may provide further insights. Interventional studies evaluating strategies to reduce the need for unplanned transitions from PD to hemodialysis are warranted because this seems to be a high-risk scenario.

Our findings have important implications for clinical practice. Nephrologists, cardiologists, and cardiac surgeons should consider our results when selecting a dialysis modality and managing patients in the perioperative period. Continuing PD patients on PD postcardiac surgery is associated with lower morbidity, reduced length of stay, and hospital charges. Protocols should be developed to optimize the care of PD patients undergoing cardiac surgery, with the goal of maintaining PD whenever possible.

## Supplementary Material

SUPPLEMENTARY MATERIAL

## Data Availability

Partial restrictions to the data and/or materials apply. Data can be obtained from the Healthcare Cost & Utilization Project's NIS database, available online at https://hcup-us.ahrq.gov/db/nation/nis/nisdbdocumentation.jsp.

## Supplemental Material

Supplemental Table 1. ICD codes cardiac surgery.

Supplemental Table 2. ICD codes CCMI.

Supplemental Table 3. Individual surgeries by modality.

Supplemental Figure 1. Prolonged ventilation subgroups.

Supplemental Figure 2. Length of stay subgroups.

Supplemental Figure 3. Hospital charges subgroups.
