# Supplementary material for: Effect of Dialysis Modality on Mortality and Complications in Cardiovascular Surgery: Insights from a National Retrospective Cohort Study
Source: Kidney360. 2025 Jan 16;6(5):784–92. doi: 10.34067/KID.0000000701 (PMC12136638; doi:10.34067/KID.0000000701)
Supplement: SUPPLEMENTARY MATERIAL [file kidney360-6-784-s001.pdf]

ASN American Society of Nephrology
JASN Journal of the American Society of Nephrology
KIDNEY360 Accessing Our World From Every Angle
CJASN Clinical Journal of the American Society of Nephrology

ASN Journal Disclosure Form

As per ASN journal policy, I have disclosed any financial relationships or commitments I have held in the past 36 months as included below. I have listed my Current Employer below to indicate there is a relationship requiring disclosure. If no relationship exists, my Current Employer is not listed.

O. El Shamy reports the following:
Employer: George Washington University; Consultancy: Outset Medical; Honoraria: Home Dialysis University ; UpToDate; and Advisory or Leadership Role: Light Line Medical Inc, Akebia.

I understand that the information above will be published within the journal article, if accepted, and that failure to comply and/or to accurately and completely report the potential financial conflicts of interest could lead to the following: 1) Prior to publication, article rejection, or 2) Post-publication, sanctions ranging from, but not limited to, issuing a correction, reporting the inaccurate information to the authors' institution, banning authors from submitting work to ASN journals for varying lengths of time, and/or retraction of the published work.

Name: Osama El Shamy
Manuscript ID: K360-2024-000962R1
Manuscript Title: Impact of Dialysis Modality on Mortality and Complications in Cardiovascular Surgery: Insights from a National Retrospective Cohort Study
Date of Completion: December 20, 2024
Disclosure Updated Date: December 20, 2024

ASN American Society of Nephrology | JASN Journal of the American Society of Nephrology | KIDNEY360 Accessing Our World From Every Angle | CJASN Clinical Journal of the American Society of Nephrology

ASN Journal Disclosure Form

As per ASN journal policy, I have disclosed any financial relationships or commitments I have held in the past 36 months as included below. I have listed my Current Employer below to indicate there is a relationship requiring disclosure. If no relationship exists, my Current Employer is not listed.

S. Hu reports the following:
Employer: Brown School of Medicine; Ownership Interest: GEHC- less than $1500; WBA- less than $2000;; Research Funding: Moderna- Lyme vaccine development (no available product being marketed); Tarsus- anti-tick medication (no available product being marketed);; and Patents or Royalties: Pending- antiphospholipid antibodies for Lyme diagnostics.

I understand that the information above will be published within the journal article, if accepted, and that failure to comply and/or to accurately and completely report the potential financial conflicts of interest could lead to the following: 1) Prior to publication, article rejection, or 2) Post-publication, sanctions ranging from, but not limited to, issuing a correction, reporting the inaccurate information to the authors' institution, banning authors from submitting work to ASN journals for varying lengths of time, and/or retraction of the published work.

Name: Susie L. Hu
Manuscript ID: K360-2024-000962R1
Manuscript Title: Impact of Dialysis Modality on Mortality and Complications in Cardiovascular Surgery: Insights from a National Retrospective Cohort Study
Date of Completion: December 19, 2024
Disclosure Updated Date: December 19, 2024

ASN American Society of Nephrology | JASN Journal of the American Society of Nephrology | KIDNEY360 Accessing Our World From Every Angle | CJASN Clinical Journal of the American Society of Nephrology

ASN Journal Disclosure Form

As per ASN journal policy, I have disclosed any financial relationships or commitments I have held in the past 36 months as included below. I have listed my Current Employer below to indicate there is a relationship requiring disclosure. If no relationship exists, my Current Employer is not listed.

J. Perl reports the following:
Employer: St. Michael's Hospital; Consultancy: Baxter Health Care Canada; Bayer, Otsuka, Fresenius Medical Care, Davita Healthcare Partners, ; LiberDi; Astra Zeneca ; Otsuka, Outset Medical; Ownership Interest: I-Ren; Research Funding: Arbor Research Collaborative For Health; AHRQ; Honoraria: Baxter healthcare usa/canada ; Davita Healthcare partners; Fresenius Medical Care; DCI; , Astra Zeneca, US Renal Care, Bayer Canada, Otsuka, Innovative Renal Care; Speakers Bureau: Baxter Healthcare; Fresenius Medical Care; and Other Interests or Relationships: Salary Support: Arbor Research Collaborative For Health; AHRQ, Stipend ISPD, ASN.

I understand that the information above will be published within the journal article, if accepted, and that failure to comply and/or to accurately and completely report the potential financial conflicts of interest could lead to the following: 1) Prior to publication, article rejection, or 2) Post-publication, sanctions ranging from, but not limited to, issuing a correction, reporting the inaccurate information to the authors' institution, banning authors from submitting work to ASN journals for varying lengths of time, and/or retraction of the published work.

Name: Jeffrey Perl
Manuscript ID: K360-2024-000962R1
Manuscript Title: mpact of Dialysis Modality on Mortality and Complications in Cardiovascular Surgery: Insights from a National Retrospective Cohort Stud
Date of Completion: December 19, 2024
Disclosure Updated Date: September 26, 2024

ASN
American Society of Nephrology
JASN
Journal of the American Society of Nephrology
KIDNEY360
Accessing Our World From Every Angle
CJASN
Clinical Journal of the American Society of Nephrology

ASN Journal Disclosure Form

As per ASN journal policy, I have disclosed any financial relationships or commitments I have held in the past 36 months as included below. I have listed my Current Employer below to indicate there is a relationship requiring disclosure. If no relationship exists, my Current Employer is not listed.

C. Raker reports the following:
Employer: Lifespan Health System; NTT Data

I understand that the information above will be published within the journal article, if accepted, and that failure to comply and/or to accurately and completely report the potential financial conflicts of interest could lead to the following: 1) Prior to publication, article rejection, or 2) Post-publication, sanctions ranging from, but not limited to, issuing a correction, reporting the inaccurate information to the authors' institution, banning authors from submitting work to ASN journals for varying lengths of time, and/or retraction of the published work.

Name: Christina A. Raker
Manuscript ID: K360-2024-000962R1
Manuscript Title: Impact of Dialysis Modality on Mortality and Complications in Cardiovascular Surgery: Insights from a National Retrospective Cohort Study
Date of Completion: December 19, 2024
Disclosure Updated Date: May 11, 2024

ASN American Society of Nephrology
JASN Journal of the American Society of Nephrology
KIDNEY360 Accessing Our World From Every Angle
CJASN Clinical Journal of the American Society of Nephrology

ASN Journal Disclosure Form

As per ASN journal policy, I have disclosed any financial relationships or commitments I have held in the past 36 months as included below. I have listed my Current Employer below to indicate there is a relationship requiring disclosure. If no relationship exists, my Current Employer is not listed.

A. Shah reports the following:
Employer: Brown Physicians Inc; Consultancy: Otsuka, Calliditas; and Advisory or Leadership Role: American College of Physicians Rhode Island Chapter Governors Advisory Council ; American Society of Nephrology Policy and Advocacy Committee; Renal Physicians Association Government Affairs Committee; Renal Physicians Association Policy Advocacy Leadership Steering Committee.

I understand that the information above will be published within the journal article, if accepted, and that failure to comply and/or to accurately and completely report the potential financial conflicts of interest could lead to the following: 1) Prior to publication, article rejection, or 2) Post-publication, sanctions ranging from, but not limited to, issuing a correction, reporting the inaccurate information to the authors' institution, banning authors from submitting work to ASN journals for varying lengths of time, and/or retraction of the published work.

Name: Ankur Shah
Manuscript ID: K360-2024-000962R1
Manuscript Title: Impact of Dialysis Modality on Mortality and Complications in Cardiovascular Surgery: Insights from a National Retrospective Cohort Study
Date of Completion: January 6, 2025
Disclosure Updated Date: November 5, 2024
