# Supplementary material for: Effect of Dialysis Modality on Mortality and Complications in Cardiovascular Surgery: Insights from a National Retrospective Cohort Study
Source: Kidney360. 2025 Jan 16;6(5):784–92. doi: 10.34067/KID.0000000701 (PMC12136638; doi:10.34067/KID.0000000701)
Supplement: SUPPLEMENTARY MATERIAL [file kidney360-6-784-s002.pdf]

Supplemental Table 1 ICD Codes Cardiac Surgery

**CABG**	**Variations**
0210xxx	083, 088, 089, 08C, 08F, 08W, 093, 098, 099, 09C, 09F, 09W, 0A3, 0A8, 0A9, 0AC, 0AF, 0AW, 0J3, 0J8, 0J9, 0JC, 0JF, 0JW, 0K3, 0K8, 0K9, 0KC, 0KF, 0KW, 0Z3, 0Z8, 0Z9, 0ZC, 0ZF, 444, 483, 488, 489, 48C, 48F, 48W, 493, 498, 499, 49C, 49F, 49W, 4A3, 4A8, 4A9, 4AC, 4AF, 4AW, 4D4, 4J3, 4J8, 4J9, 4JC, 4JF, 4JW, 4K3, 4K8, 4K9, 4KC, 4KF, 4KW, 4Z3, 4Z8, 4Z9, 4ZC, 4ZF
0211xxx	083, 088, 089, 08C, 08F, 08W, 093, 098, 099, 09C, 09F, 09W, 0A3, 0A8, 0A9, 0AC, 0AF, 0AW, 0J3, 0J8, 0J9, 0JC, 0JF, 0JW, 0K3, 0K8, 0K9, 0KC, 0KF, 0KW, 0Z3, 0Z8, 0Z9, 0ZC, 0ZF, 444, 483, 488, 489, 48C, 48F, 48W, 493, 498, 499, 49C, 49F, 49W, 4A3, 4A8, 4A9, 4AC, 4AF, 4AW, 4D4, 4J3, 4J8, 4J9, 4JC, 4JF, 4JW, 4K3, 4K8, 4K9, 4KC, 4KF, 4KW, 4Z3, 4Z8, 4Z9, 4ZC, 4ZF
0212xxx	083, 088, 089, 08C, 08F, 08W, 093, 098, 099, 09C, 09F, 09W, 0A3, 0A8, 0A9, 0AC, 0AF, 0AW, 0J3, 0J8, 0J9, 0JC, 0JF, 0JW, 0K3, 0K8, 0K9, 0KC, 0KF, 0KW, 0Z3, 0Z8, 0Z9, 0ZC, 0ZF, 444, 483, 488, 489, 48C, 48F, 48W, 493, 498, 499, 49C, 49F, 49W, 4A3, 4A8, 4A9, 4AC, 4AF, 4AW, 4D4, 4J3, 4J8, 4J9, 4JC, 4JF, 4JW, 4K3, 4K8, 4K9, 4KC, 4KF, 4KW, 4Z3, 4Z8, 4Z9, 4ZC, 4ZF
0213xxx	083, 088, 089, 08C, 08F, 08W, 093, 098, 099, 09C, 09F, 09W, 0A3, 0A8, 0A9, 0AC, 0AF, 0AW, 0J3, 0J8, 0J9, 0JC, 0JF, 0JW, 0K3, 0K8, 0K9, 0KC, 0KF, 0KW, 0Z3, 0Z8, 0Z9, 0ZC, 0ZF, 444, 483, 488, 489, 48C, 48F, 48W, 493, 498, 499, 49C, 49F, 49W, 4A3, 4A8, 4A9, 4AC, 4AF, 4AW, 4D4, 4J3, 4J8, 4J9, 4JC, 4JF, 4JW, 4K3, 4K8, 4K9, 4KC, 4KF, 4KW, 4Z3, 4Z8, 4Z9, 4ZC, 4ZF
**Valve**	**Variations**
024xxxx	F07J, F08J, F0JJ, F0KJ, G072, G082, G0J2, G0K2, J072, J082, J0J2, J0K2
025xxZZ	F0, F4, G0, G4, H0, H4, J0, J4
027xxxx	F04Z, F0DZ, F0ZZ, F44Z, F4DZ, F4ZZ, G04Z, G0DZ, G0ZZ, G44Z, G4DZ, G4ZZ, H04Z, H0DZ, H0ZZ, H44Z, H4DZ, H4ZZ, J04Z, J0DZ, J0ZZ, J44Z, J4DZ, J4ZZ
02Bxxxx	F0ZX, F0ZZ, F4ZX, F4ZZ, G0ZX, G0ZZ, G4ZX, G4ZZ, H0ZX, H0ZZ, H4ZX, H4ZZ, J0ZX, J0ZZ, J4ZX, J4ZZ
02CxxZZ	F0, F4, G0, G4, H0, H4, J0, J4
02LHxxx	0CZ, 0DZ, 0ZZ, 4CZ, 4DZ, 4ZZ
02NxxZZ	F0, F4, G0, G4, H0, H4, J0, J4
02QxxZx	F0ZJ, F0ZZ, F4ZJ, F4ZZ, G0ZE, G0ZZ, G4ZE, G4ZZ, H0ZZ, H4ZZ, J0ZG, J0ZZ, J4ZG, J4ZZ
02Rxxxx	F07Z, F08N, F08Z, F0JZ, F0KZ, F47Z, F48N, F48Z, F4JZ, F4KZ, G07Z, G08Z, G0JZ, G0KZ, G47Z, G48Z, G4JZ, G4KZ, H07Z, H08Z, H0JZ, H0KZ, H47Z, H48Z, H4JZ, H4KZ, J07Z, J08Z, J0JZ, J0KZ, J47Z, J48Z, J4JZ, J4KZ
02THxZZ	0, 4
02Uxxxx	F07J, F07Z, F08J, F08Z, F0JJ, F0JZ, F0KJ, F0KZ, F47J, F47Z, F48J, F48Z, F4JJ, F4JZ, F4KJ, F4KZ, G07E, G07Z, G08E, G08Z, G0JE, G0JZ, G0KE,

	G0KZ, G47E, G47Z, G48E, G48Z, G4JE, G4JZ, G4KE, G4KZ, H07Z, H08Z, H0JZ, H0KZ, H47Z, H48Z, H4JZ, H4KZ, J07G, J07Z, J08G, J08Z, J0JG, J0JZ, J0KG, J0KZ, J47G, J47Z, J48G, J48Z, J4JG, J4JZ, J4KG, J4KZ
02VGxZZ	0, 4
02Wxxxx	F07Z, F08Z, F0JZ, F0KZ, F47Z, F48Z, F4JZ, F4KZ, G07Z, G08Z, G0JZ, G0KZ, G47Z, G48Z, G4JZ, G4KZ, H07Z, H08Z, H0JZ, H0KZ, H47Z, H48Z, H4JZ, H4KZ, J07Z, J08Z, J0JZ, J0KZ, J47Z, J48Z, J4JZ, J4KZ
X2RFxxx	032, 432

Supplemental Table 2 ICD Codes CCMI

Medical Condition or Procedure	ICD-10 Codes
Congestive Heart Failure (CHF)	I09.9, I11.0, I13.0, I13.2, I25.5, I42.0, I42.5-I42.9, I43.x, I50.x, P29.0
Cerebrovascular Disease	G45.x, G46.x, H34.0, I60.x-I69.x
Diabetes with and without Chronic Complication	E10.0, E10.1, E10.6, E10.8, E10.9, E11.0, E11.1, E11.6, E11.8, E11.9, E12.0, E12.1, E12.6, E12.8, E12.9, E13.0, E13.1, E13.6, E13.8, E13.9, E14.0, E14.1, E14.6, E14.8, E14.9, E10.2-E10.5, E10.7, E11.2-E11.5, E11.7, E12.2-E12.5, E12.7, E13.2-250.4-250.7, E13.5, E13.7, E14.2-E14.5, E14.7
Malignancy	C00.-C26.x, C30.x-C34.x, C37.x-C41.x, C43.x, C45.x-058.x, C60.x-C76.x, C81.x-C85.x, C88.x, C90.x-C97.x

Supplementary Table 3
Individual Surgeries by modality

Outcome	HD	PD	Unadjusted	Adjusted*
CABG only				
Mortality	1050 (5.7%)	65 (4.1%)	0.69 (0.39, 1.24)	0.82 (0.45, 1.49)
Prolonged Ventilation	1215 (6.7%)	60 (3.8%)	0.55 (0.30, 0.99)	0.63 (0.34, 1.15)
LOS (Mean/SD)	14.6 (13.2)	12.2 (8.0)	0.83 (0.77, 0.89)	0.89 (0.83, 0.95)
Total Charges (Mean/SD)	343943 (323819)	275850 (199561)	-68092 (-92848, -43336)	-56432 (-81240, -31625)
Valve Only				
**Outcome**	**HD**	**PD**	**Unadjusted**	**Adjusted***
Mortality	600 (9.7%)	10 (3.6%)	0.35 (0.09, 1.47)	0.29 (0.07, 1.19)
Prolonged Ventilation	890 (14.3%)	20 (7.3%)	0.47 (0.17, 1.32)	0.35 (0.12, 1.06)
LOS (Mean/SD)	22.3 (24.6)	18.3 (14.2)	0.82 (0.66, 1.02)	0.82 (0.65, 1.02)
Total Charges (Mean/SD)	532554 (645482)	436799 (273273)	-95755 (-181139, -10371)	-128464 (-226992, -29936)
Combined CABG/Valve				
**Outcome**	**HD**	**PD**	**Unadjusted**	**Adjusted***
Mortality	520 (14.6%)	20 (7.3%)	0.46 (0.16, 1.30)	0.43 (0.14, 1.27)
Prolonged Ventilation	625 (17.5%)	20 (7.3%)	0.37 (0.13, 1.05)	0.36 (0.11, 1.13)
LOS (Mean/SD)	20.8 (17.1)	17.1 (11.2)	0.82 (0.69, 0.98)	0.81 (0.68, 0.96)
Total Charges (Mean/SD)	515195 (406899)	353890 (229871)	-161305 (-227202, -95407)	(-169790, -243447, -96133)

*Adjusted for age, gender, race, Charlson comorbidity index, frailty, hypertension, diabetes, cancer, hospital size, location, teaching status, region, and primary payer. Odds ratios reported for mortality and prolonged ventilation, incidence rate ratios for LOS

Supplemental Figure 1 Prolonged Ventilation Subgroups

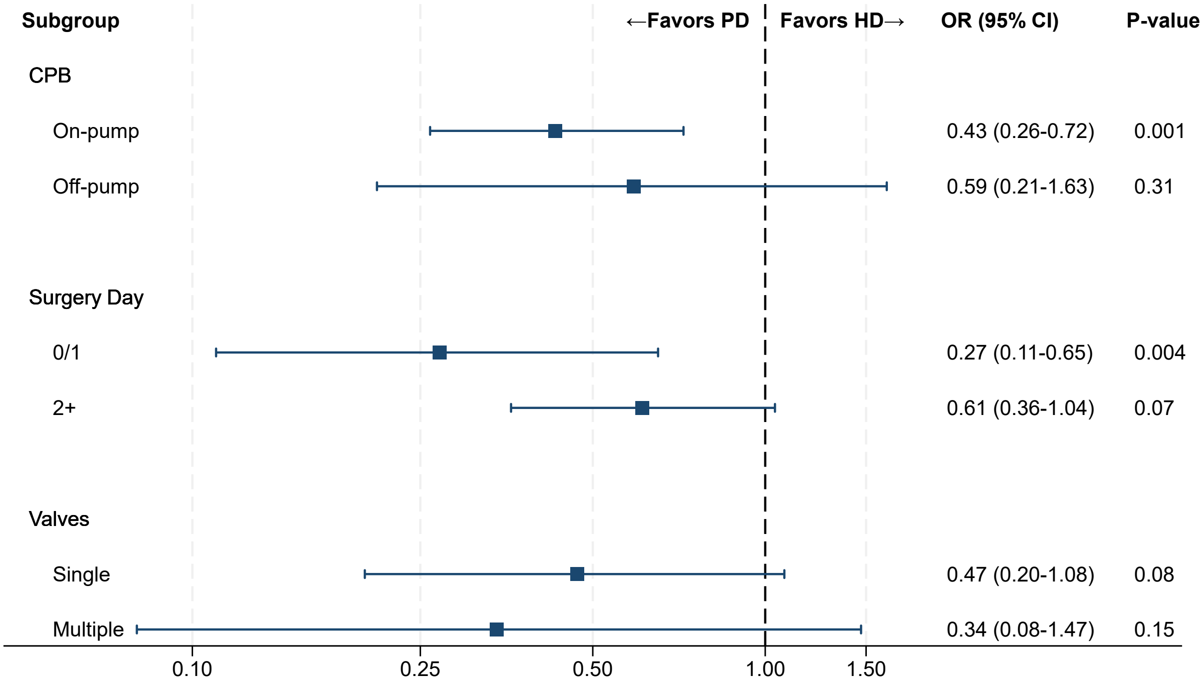

*PD: Peritoneal dialysis; CPB: Cardiopulmonary Bypass; OR: Odds ratio; IRR: Incidence rate ratio; MD: Mean difference; CI: Confidence interval

Supplemental Figure 2 Length of Stay Subgroups

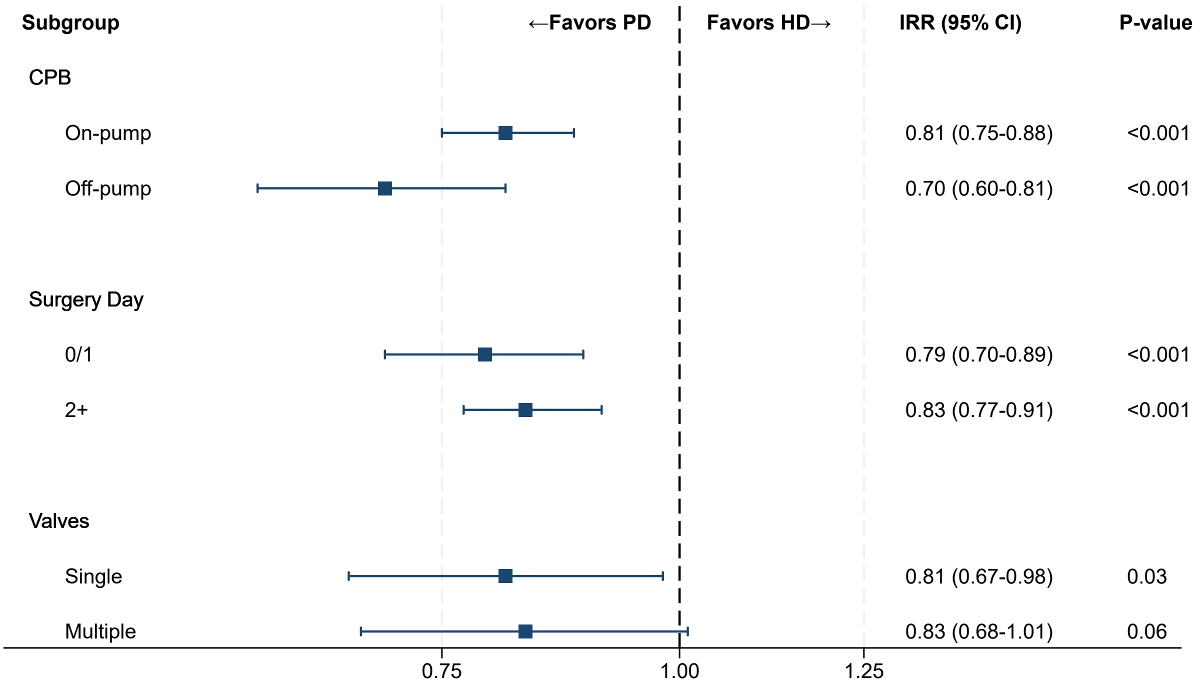

*PD: Peritoneal dialysis; CPB: Cardiopulmonary Bypass; OR: Odds ratio; IRR: Incidence rate ratio; MD: Mean difference; CI: Confidence interval

Supplemental Figure 3 Hospital Charges Subgroups

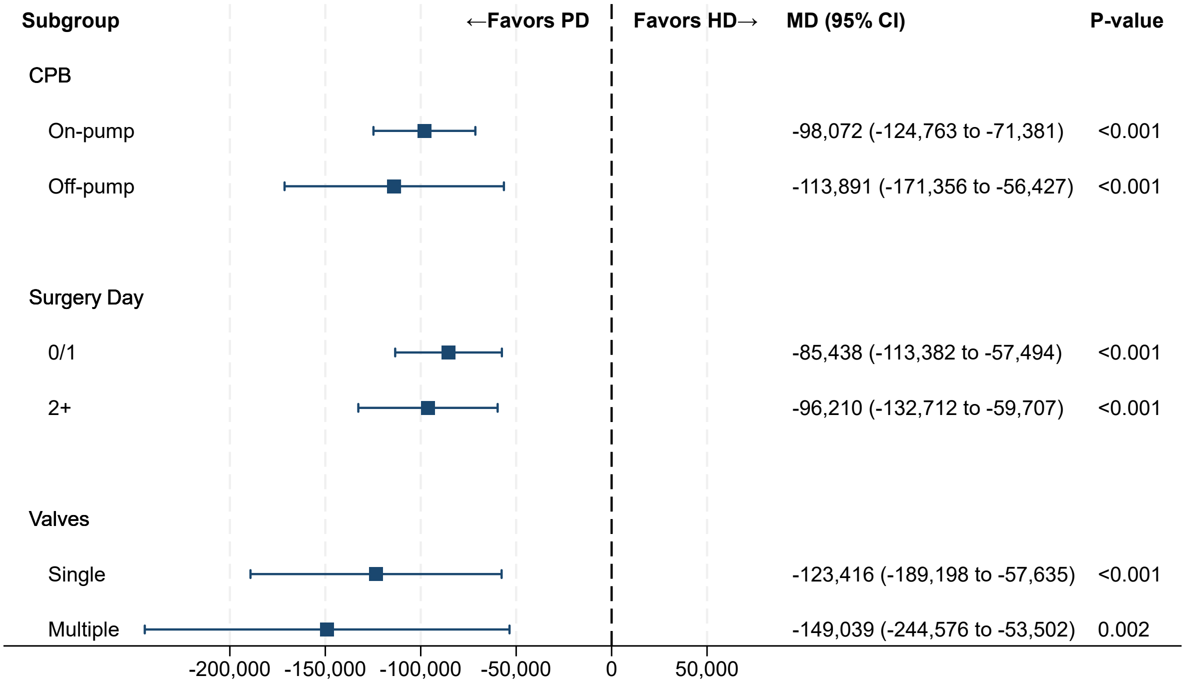

*PD: Peritoneal dialysis; CPB: Cardiopulmonary Bypass; OR: Odds ratio; IRR: Incidence rate ratio; MD: Mean difference; CI: Confidence interval
